# Assessing CB1 and CB2 receptor agonist antinociception while exploring sex differences and 5-HT1A-dependent mechanisms

**DOI:** 10.3389/fpain.2026.2005000

**Published:** 2026-10-26

**Authors:** Robert C. Barnes, Hannah Quick, Dakota Robison, Aric F. Logsdon, Josée Guindon

**Affiliations:** 1 Department of Pharmacology and Neuroscience, Texas Tech University Health Sciences Center, Lubbock, TX, United States; 2 Center of Excellence for Translational Neuroscience and Therapeutics, Texas Tech University Health Sciences Center, Lubbock, TX, United States; 3 Brain Drug Discovery Center, Texas Tech University Health Sciences Center, Amarillo, TX, United States

**Keywords:** 5-HT1A, acute pain, cannabinoid, cannabinoid receptor 1 (CB1), cannabinoid receptor 2 (CB2), formalin, inflammatory pain, sex differences

## Abstract

**Introduction:**

Inflammatory pain is a key component of both acute and chronic pain conditions that, when pathological, significantly decrease patient quality of life. There has been a recent interest in the use of cannabinoid-based compounds for analgesia and recent research has discovered functional connections between the CB_2_ and 5-HT_1A_ receptors.

**Methods:**

Therefore, we investigated the efficacy of CB_1_-selective agonism (ACEA) and CB_2_-selective agonism (AM1241), and their potential reliance on the 5-HT_1A_ receptor, in the formalin inflammatory pain model using female and male adult C57BL/6J mice. Potential dose- and sex-differences were assessed for ACEA (at 0.1-2.5 mg/kg) and AM1241 (at 1-10 mg/kg) in the formalin test. Subsequent experiments were conducted using either ACEA (0.5 mg/kg) or AM1241 (10 mg/kg), alone or with inhibition of 5-HT_1A_ via WAY-100635, in both the formalin model of inflammatory pain and the non-nociceptive components of the cannabinoid tetrad test (open field, rotarod, rectal temperature).

**Results:**

ACEA provided significant pain relief at 0.5 mg/kg in female mice and at 0.5 and 0.75 mg/kg in male mice, with significant sex differences observed. ACEA (0.5 mg/kg) antinociception was unaltered by 5-HT_1A_ inhibition while AM1241 (10 mg/kg) antinociception was completely prevented in the acute phase by 5-HT_1A_ inhibition. Neither ACEA nor AM1241 significantly altered performance in the open field, rotarod, or rectal temperature assessments.

**Discussion:**

These results demonstrate the antinociceptive benefit of CB1 and CB_2_ agonism in a murine model of inflammatory pain while demonstrating further evidence of a functional link between the CB_2_ and 5-HT_1A_ receptors. Further studies are needed to investigate the mechanism behind this connection and its potential clinical significance.

## Introduction

Inflammatory pain is a key component of both acute traumatic pain and chronic rheumatic disease and is a consequence of peripheral and central tissue hypersensitization (1–3). This hypersensitization results in increased perception of and emotional response to noxious stimuli (4). In non-pathological states, this response increases the perception of pain while damaged tissues are repaired to prevent reinjury and speed recovery (5, 6). However, when acute inflammation fails to resolve, it results in chronic inflammatory pain that contributes to multiple chronic pain conditions and negatively impacts patient quality of life (7). One such condition is chronic rheumatic disease, which affects one-in-five US adults, significantly reduces their quality of life, and is refractory to all available treatment options in 21% of cases (8–11). Though most pain research is male focused, female sex has repeatedly been associated as a risk factor for the development of chronic pain (12–16). Murine models, frequently used to assess pain, reveal similar sex differences in models of both acute and chronic pain (17). Current pain management guidelines eschew opioids while favoring a multimodal approach to pain that combines nonsteroidal anti-inflammatory drugs, anticonvulsants, and antidepressants (18). However, it is possible that this approach will be revisited as prolonged anticonvulsant (gabapentin or pregabalin) use for pain management has recently been associated with an increased risk for Alzheimer's disease (19, 20).

Cannabis has been used as an analgesic for millennia and there has generally been a renewed interest in the antinociceptive potential of cannabinoid compounds, either as an alternative or adjuvant treatment option, for poorly managed chronic pain conditions (21, 22). Previous preclinical research into their antinociceptive efficacy have shown significant pain relief and significant sex differences in their effects (23–25). Both the phytocannabinoid Δ9-tetrahydrocannabinol (Δ9-THC) and the endocannabinoid system act primarily through activation of cannabinoid receptor 1 (CB_1_) and cannabinoid receptor 2 (CB_2_) (26). Agonists targeting either the CB_1_ (24, 27) or CB_2_ (28–30) have proven to provide significant antinociceptive benefit in preclinical models and both receptors have been implicated in inflammatory hypersensitivity (31). Chronic use of cannabis or cannabinoids for antinociception is limited by the development of tolerance at CB_1_, though not CB_2_, receptors (32–35).

The serotonin 1A receptor (5-HT_1A_) has recently been identified as producing antinociceptive and anti-depressive effects when activated and cannabidiol, among other phytocannabinoids, has been found to produce positive allosteric modulation at this receptor to produce these effects (25, 36–41). The 5-HT_1A_ receptor has been further connected to the endocannabinoid system through the finding that there are functional connections between 5-HT_1A_ and CB_2_ (42, 43). However, it is still unclear the significance of these functional connections in relation to the antinociceptive and anti-inflammatory effects of cannabinoid receptor activation and whether the CB_1_ receptor bears similar functional connections to 5-HT_1A_.

To address this knowledge gap, our study investigated the efficacy of selective agonism of CB_1_ (ACEA) or AM1241 (CB_2_) at varying doses in the formalin model of inflammatory pain, using C57BL/6J male and female mice. All subsequent experiments were conducted using the doses of ACEA (0.5 mg/kg) or AM1241 (10 mg/kg) that proved most effective in producing antinociception in the formalin model of inflammatory pain. The reliance on the 5-HT_1A_ receptor for the antinociceptive efficacy of CB1 agonism (ACEA) and CB2 agonism (AM1241) was then investigated using 5-HT_1A_ antagonism prior to cannabinoid receptor activation, revealing that the antagonism of 5-HT_1A_ prevented CB2, but not CB1, mediated acute phase antinociception. Finally, the effects of these cannabinoid agonists, both with and without prior inhibition of 5-HT_1A_, were investigated in murine models of locomotion (open field), motor coordination (rotarod), and temperature (rectal temperature), with no significant differences being noted. These findings emphasize the need for further studies investigating the involvement of 5-HT_1A_ in CB_2_-mediated effects.

## Materials and methods

### Animals

Male and female C57BL/6J mice were obtained from Jackson Laboratories (Bar Harbor, Maine) and used for all experiments. All animal care and experimental procedures used in this study were approved of by the Institutional Animal Care and Use Committee (IACUC) of the Texas Tech University Health Sciences Center and conducted in accordance with guidelines found in the Guide for the Care and Use of Laboratory Animals and published by the National Institutes of Health (NIH) (44). Mice were housed under 12:12 h light-dark cycle (lights on 06:00, lights off 18:00) with *ad libitum* access to standard mouse chow with 19.8% protein.

### Drugs

Arachidonoyl 2′-chloroethylamide (ACEA; CB_1_ selective agonist) and AM1241 (CB_2_ selective agonist) were obtained from Cayman Chemical (Ann Arbor, MI, USA). WAY-100635 (5-HT_1A_ selective antagonist) was obtained from Sigma-Aldrich (St. Louis, MO, USA). Formalin was obtained from Thermo Fisher Scientific (Waltham, MA, USA). ACEA was used at a variety of doses (0.1, 0.25, 0.5, 0.75, 1.0, and 2.5 mg/kg) to determine the optimal dose for antinociception during the formalin test, with doses within that range having previously proven effective in other murine models of pain (24, 45). Similarly, AM1241 was explored at a variety of doses (1, 3, and 10 mg/kg) to determine the optimal dose for use in the formalin test, with these doses having previously proven effective in other murine pain models (24, 46, 47). WAY-100635 (0.1 mg/kg) was prepared at doses previously shown to be effective (40). WAY-100635 was dissolved in normal saline (0.9% NaCl in water) while ACEA and AM1241 were dissolved in a vehicle solution consisting of dimethylsulfoxide, ethanol, Tween80, and normal saline at a ratio of 1:1:1:17. This vehicle solution was used as control for all experiments. All vehicle solutions and drugs were prepared fresh on the day of experimentation and administered intraperitoneally (i,p.) in a single volume of 10 µL/g of body weight. ACEA, AM1241, WAY-100635, or vehicle were administered 30 min prior to the start of either formalin testing or rectal temperature/open field/rotarod testing, depending on the cohort. Mice that received both WAY-100635 and ACEA or AM1241 received the injection of WAY-100635 30 min prior to their injection with either ACEA or AM1241; control mice for these experiments received a single injection of vehicle solution 30 min prior to the start of behavioral testing.

### Formalin test

The formalin test was performed by injecting dilute formalin solution (2.5%; 10 µL) into the right hind paw of each mouse, with mice being observed and pain behavior scored for one hour (48, 49). Mice were observed on a glass observation table while in a 10 cm × 10 cm × 10 cm clear plexiglass house, with viewing aided by a mirror situated underneath at a 45^o^ angle. The formalin test consists of two phases: the acute phase (phase 1; 0–15 min), mediated by transient receptor potential ankyrin 1 (TRPA1) peripheral nociceptors (50), and the inflammatory phase (phase 2; 15–60 min), mediated by inflammatory-mediated peripheral and central sensitization (51, 52). Pain behavior is grouped into 4 categories: no pain behavior, favoring (injected paw has limited weight bearing), lifting (injected paw held from contact with any surface), and rapid licking/shaking/biting. This pain behavior is divided into 5-minute intervals and the composite pain score (CPS) is calculated using the CPS-weighted scores technique (CPS-WST 0, 1, 2) (53). The trapezoidal rule is used to calculate the area under the curve (AUC) for both the acute and inflammatory phases of formalin pain (54). All mice were humanely euthanized by carbon monoxide inhalation after completion of the formalin test.

### Triad test

A separate cohort of male and female mice were evaluated for the potential effects of the tested cannabinoid compounds (ACEA 0.5 mg/kg, AM1241 10 mg/kg) on the non-nociceptive components of the cannabinoid tetrad test (locomotion, motor coordination, and temperature).

### Open field (locomotion)

The open field test was used to assess both locomotion (total movement) and anxiety phenotype, during a 5-minute trial period in an open field test arena with dimensions 49 cm × 39 cm × 21 cm (length × width × height). An overhead LED light provided uniform light at an intensity of 200 lux, as measured by a digital light meter. A Basler digital camera (Model acA 1300-60gc, Ahrensburg, Germany) and Ethovision XT 11.5 tracking software (Noldus, Lesburg, VA, USA) were used to track motion. Baseline measurements were assessed 24 h prior to any injection and the open field test was conducted 30 min, 90 min, 6 h, and 24 h after injection.

### Rotarod (motor coordination)

The accelerating rotarod test was used to assess motor coordination (latency to fall) (Model LE8205, Harvard Apparatus, Holliston, MA, USA). Mice were situated on the accelerating rotarod while it revolved at 4 rotations per minute (RPM), with the rotarod programmed to accelerate to 40 RPM over a 5-minute period. Mice received 3 days of rotarod training prior to experimental testing and each mouse received three trials per testing period with a ten-minute inter-trial interval. Baseline measurements were assessed 24 h prior to any injection and the rotarod test was conducted 30 min, 90 min, 6 h, and 24 h after injection.

### Rectal temperature (temperature)

Body temperature was assessed through use of a mouse rectal thermometer (Physitemp, Clifton, NJ, USA). Baseline measurements were assessed immediately prior to any injection and at 30 min, 90 min, 6 h, and 24 h after injection.

### Data analysis and statistics

All experiments were conducted with animals randomly assigned to experimental conditions and in a blind manner. All data was expressed as mean ± SEM. Data analysis was performed via analysis of variance (ANOVA) for repeated measures with Greenhouse–Geisser correction, one-way ANOVA, or two-way ANOVA, as appropriate. Reported degrees of freedom for all significant interactions are provided as the uncorrected values. All significant interactions were further evaluated through multiple comparison analysis with Bonferroni corrections for *post hoc* analysis. Components of total variation were settled *a priori* using multiple regression analysis (55). Analyses were performed using GraphPad Prism (version 10.6.0, GraphPad Software, San Diego, CA, USA). *P* < 0.05 was considered significant.

## Results

### ACEA provides dose- and sex-dependent antinociceptive benefit in the formalin model of inflammatory pain

Treatment of C57BL/6J female mice with ACEA (0.1, 0.25, 0.5, 0.75, 1.0, and 2.5 mg/kg i.p.) resulted in significant differences in pain behaviors between the treatments (*F*_6,35_ = 4.484, *P* = 0.002) that persisted in a time-group dependent manner (*F*_35,385_ = 2.687, *P* < 0.001) (Figure 1A). Subsequent multiple comparison analysis revealed that ACEA 0.5 mg/kg had significantly reduced pain, relative to vehicle, at 20 min (*P* = 0.02) and 25 min (*P* = 0.003), while ACEA 0.25 mg/kg had significantly reduced pain, relative to vehicle, at 25 min (*P* = 0.04) (Figure 1A). Analysis of the area under the curve (AUC) in the acute phase of pain revealed no significant differences between the treatments (*F*_6,35_ = 1.540, *P* = 0.19) (Figure 1B). In the inflammatory phase, AUC analysis revealed significant differences between the treatments (*F*_6,25_ = 4.695, *P* = 0.001), with subsequent *post-hoc* analysis demonstrating that the 0.5 mg/kg dose of ACEA provided significant antinociceptive benefit in the assessed female mice (Figure 1C).

**Figure 1:**
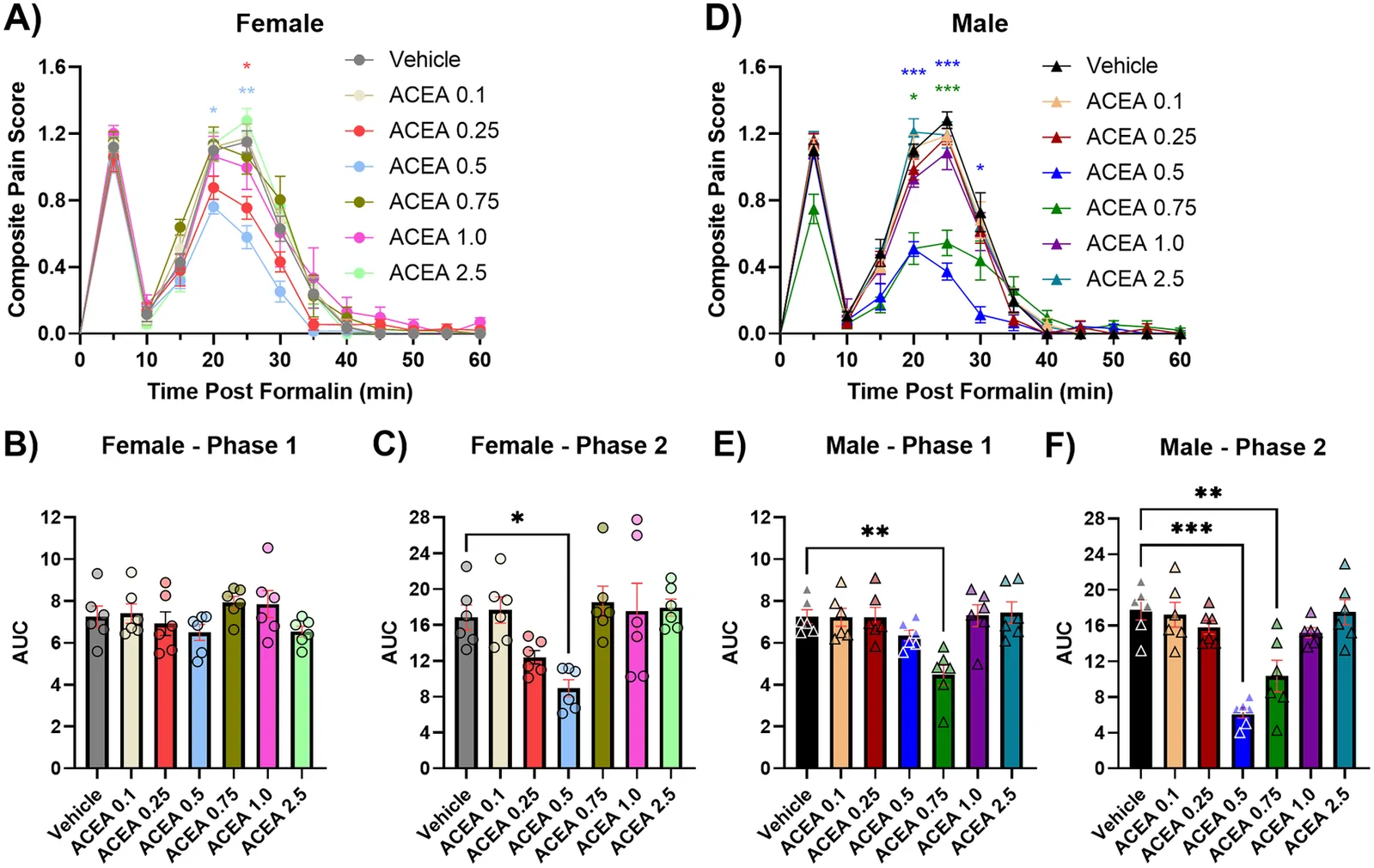
Dose- and sex-dependent antinociceptive effects of ACEA in the formalin test. ACEA suppressed formalin-induced pain behavior in both female **(A)** and male **(D)** C57BL/6J mice. In female mice, ACEA failed to suppress pain during the Acute Phase **(B)** while the 0.5 mg/kg dose of ACEA suppressed pain during the Inflammatory Phase **(C)**. In male mice, the 0.75 mg/kg dose of ACEA suppressed pain during the Acute Phase **(E)** and both the 0.5 and 0.75 mg/kg doses of ACEA suppressed pain during the Inflammatory Phase **(F)**. Significant sex differences were noted in both the acute and inflammatory phases. Data is expressed as mean ± SEM (*n* = 6 per group). Repeated measures one-way ANOVA with Greenhouse–Geisser correction used to calculate significance for **(A)** and **(D)**. One-way ANOVA with Bonferroni *post-hoc* used to calculate significance for **(B)**, **(C)**, **(E)**, and **(F)**. Relative to vehicle: *P < 0.05, **P < 0.01, ***P < 0.001.

Treatment of C57BL/6J male mice with ACEA (0.1, 0.25, 0.5, 0.75, 1.0, and 2.5 mg/kg i.p.) resulted in significant differences in pain behaviors between the treatments (*F*_6,35_ = 12.24, *P* < 0.001) that persisted in a time-group dependent manner (*F*_35,38_ = 2.982, *P* < 0.001) (Figure 1D). Multiple comparison analysis revealed that the 0.5 mg/kg dose of ACEA significantly reduced pain, relative to vehicle, at 20 through 25 min (*P* < 0.001) and at 30 min (*P* = 0.050) while the 0.75 mg/kg dose similarly reduced pain at 20 (*P* = 0.02) and 25 min (*P* < 0.001) (Figure 1D). AUC analysis revealed significant differences in the acute phase of pain (*F*_6,35_ = 5.713, *P* < 0.001) with the 0.75 mg/kg dose of ACEA significantly reducing pain, relative to vehicle, in this phase (*P* = 0.002) (Figure 1E). Similarly, AUC analysis of the inflammatory phase revealed significant differences between the treatments (*F*_6,35_ = 14.00, *P* < 0.001) with both 0.5 mg/kg (*P* < 0.001) and 0.75 mg/kg (*P* = 0.002) reducing pain, relative to vehicle (Figure 1F).

Potential sex differences in the antinociceptive efficacy of ACEA (0.1, 0.25, 0.5, 0.75, 1.0, and 2.5 mg/kg i.p.) in C57BL/6J mice was investigated through Two-Way Repeated Measures ANOVA analysis, with significant differences noted between the treatments (*F*_66,770_ = 2.841, *P* < 0.001). Pairwise comparisons revealed sex differences in the antinociceptive efficacy of ACEA 0.25 mg/kg at 25 min (*P* < 0.001), ACEA 0.5 mg/kg at 20 min (*P* = 0.020), and ACEA 0.75 mg/kg at 5 min (*P* < 0.001), 15 min (*P* < 0.001), 20 min (*P* < 0.001), 25 min (*P* < 0.001), and 30 min (*P* = 0.013). When analyzing the AUC of the acute phase, significant sex differences were noted (*F*_6,70_ = 4.717, *P* < 0.001), with subsequent pairwise comparisons revealing that male mice had significantly greater antinociceptive response to the 0.75 mg/kg dose of ACEA compared to female mice (*P* < 0.001) (Supplementary Figure S1A). In the inflammatory phase, AUC analysis revealed significant sex differences (*F*_6,70_ = 3.189, *P* = 0.008), with the 0.75 mg/kg dose of ACEA providing greater antinociceptive benefit in male mice (*P* < 0.001) (Supplementary Figure S1B). As 0.5 mg/kg was the only dose to produce antinociception in both female and male mice, all subsequent experiments involving ACEA were performed at that dose.

### AM1241 provides dose-dependent antinociceptive benefit in the formalin model of inflammatory pain

Treatment of C57BL/6J female mice with vehicle or AM1241 (1, 3, or 10 mg/kg i.p.) resulted in significant differences between the treatments (*F*_3,20_ = 62.97, *P* < 0.001) that persisted in a time-group dependent manner (*F*_33,220_ = 3.595, *P* = 0.004) (Figure 2A). Multiple comparison analysis revealed that AM1241 provided significant antinociception relative to vehicle at 1 mg/kg (at 25 min, *P* = 0.003), 3 mg/kg (at 5 min, *P* = 0.02; at 25 min, *P* < 0.001; at 30 min, *P* = 0.008), and 10 mg/kg (at 5 min, *P* = 0.006; at 25 min, *P* < 0.001; at 30 min, *P* = 0.004) (Figure 2A). AUC analysis in the acute phase of formalin-induced pain revealed significant differences between the treatments (*F*_3,20_ = 16.35, *P* < 0.001), with multiple comparison analysis revealing significant antinociception, relative to vehicle, induced by both the 3 mg/kg (*P* < 0.001) and 10 mg/kg (*P* < 0.001) doses of AM1241 (Figure 2B). Furthermore, there was significant antinociception relative to the 1 mg/kg dose for both the 3 mg/kg (*P* = 0.04) and 10 mg/kg (*P* = 0.005) doses of AM1241 (Figure 2B). AUC analysis in the inflammatory phase revealed significant differences between the treatments (*F*_3,20_ = 47.47, *P* < 0.001), with multiple comparison analysis revealing significantly reduced pain, relative to vehicle, following administration of AM1241 at 1 mg/kg (*P* < 0.001), 3 mg/kg (*P* < 0.001), and 10 mg/kg (*P* < 0.001) (Figure 2C). Additionally, there was significant antinociception relative to the 1 mg/kg dose for both the 3 mg/kg (*P* = 0.04) and 10 mg/kg (*P* < 0.001) doses of AM1241 (Figure 2C).

**Figure 2:**
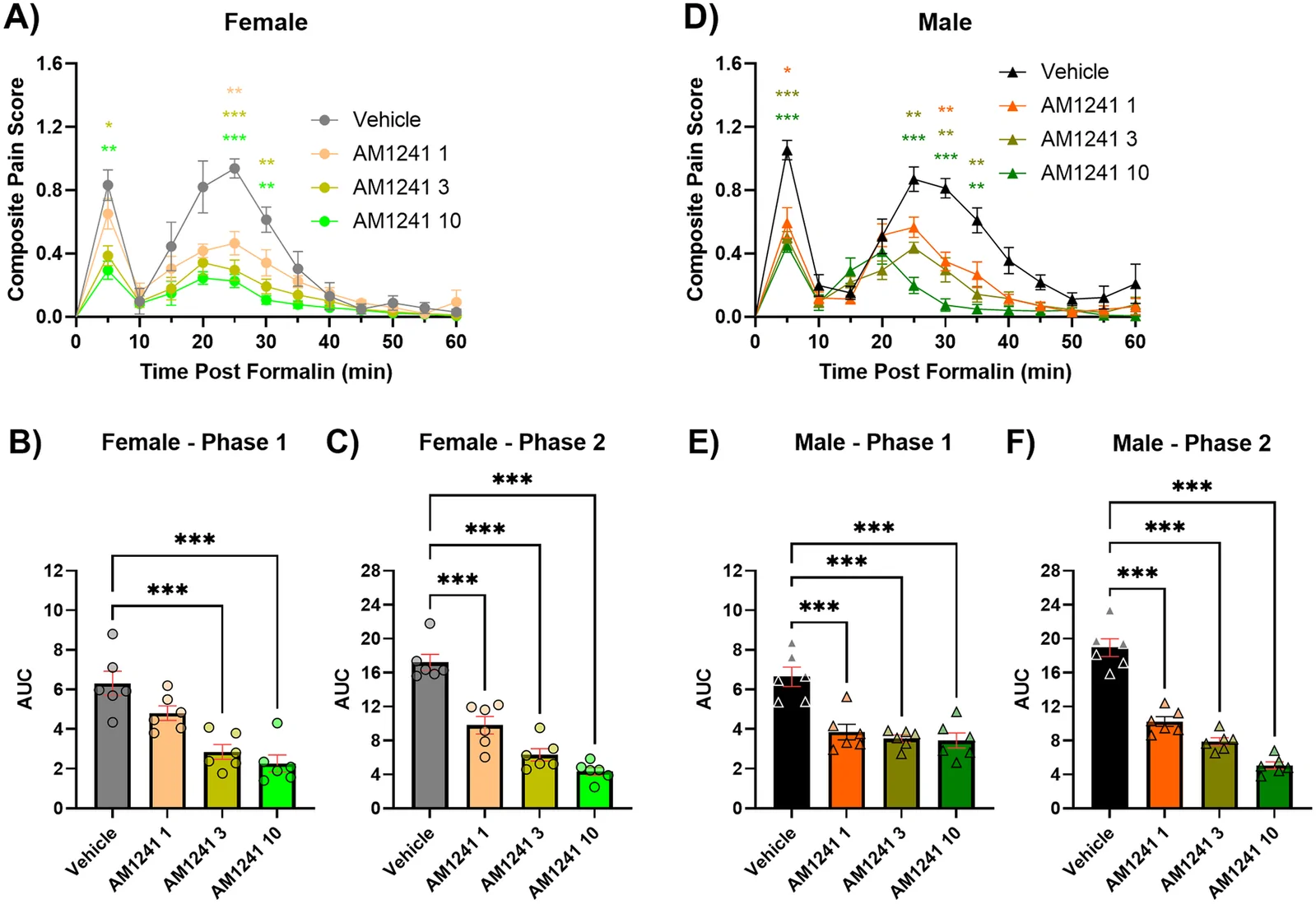
Dose-dependent antinociceptive effects of AM1241 in the formalin test. AM1241 suppressed formalin-induced pain behavior in both female **(A)** and male **(D)** C57BL/6J mice. In female mice, AM1241 at 3 and 10 mg/kg suppressed pain during the Acute Phase **(B)** while all three doses (1, 3, and 10 mg/kg) suppressed pain during the Inflammatory Phase **(C)**. In male mice, AM1241 suppressed pain at all three doses (1, 3, and 10 mg/kg) during both the Acute Phase **(E)** and Inflammatory Phase **(F)**. Data is expressed as mean ± SEM (*n* = 6 per group). Repeated measures one-way ANOVA with Greenhouse–Geisser correction used to calculate significance for **(A)** and **(D)**. One-way ANOVA with Bonferroni *post-hoc* used to calculate significance for **(B)**, **(C)**, **(E)**, and **(F)**. Relative to vehicle: *P < 0.05, **P < 0.01, ***P < 0.001.

Treatment of C57BL/6J male mice with vehicle or AM1241 (1, 3, or 10 mg/kg i.p.) resulted in significant differences between the treatments (*F*_3,20_ = 83.96, *P* < 0.001) that persisted in a time-group dependent manner (*F*_33,220_ = 4.869, *P* < 0.001) (Figure 2D). Multiple comparison analysis revealed that AM1241 provided significant antinociception relative to vehicle at 1 mg/kg (at 5 min, *P* = 0.02; at 30 min, *P* = 0.002), 3 mg/kg (at 5 min, *P* < 0.001; at 25 min, *P* = 0.009; at 30 min, *P* = 0.002; at 35 min, *P* = 0.005), and 10 mg/kg (at 5 min, *P* < 0.001; at 25 min, *P* < 0.001; at 30 min, *P* < 0.001; at 35 min, *P* = 0.002) (Figure 2D). AUC analysis in the acute phase of formalin-induced pain revealed significant differences between the treatments (*F*_3,20_ = 16.30, *P* < 0.001), with multiple comparison analysis revealing significant antinociception, relative to vehicle, induced by the 1 mg/kg (*P* < 0.001), 3 mg/kg (*P* < 0.001), and 10 mg/kg (*P* < 0.001) doses of AM1241 (Figure 2E). AUC analysis in the inflammatory phase revealed significant differences between the treatments (*F*_3,20_ = 78.78, *P* < 0.001), with multiple comparison analysis revealing significantly reduced pain, relative to vehicle, following administration of AM1241 at 1 mg/kg (*P* < 0.001), 3 mg/kg (*P* < 0.001), and 10 mg/kg (*P* < 0.001) (Figure 2F). Additionally, the 10 mg/kg dose of AM1241 provided significant antinociception relative to both the 1 mg/kg (*P* < 0.001) and 3 mg/kg (*P* = 0.050) doses of AM1241 (Figure 2F).

Potential sex differences in the antinociceptive efficacy of AM1241 at 1 mg/kg, 3 mg/kg, and 10 mg/kg were assessed through Two-Way Repeated Measures ANOVA analysis and were found to be non-significant (*F*_33,440_ = 1.611, *P* = 0.102). When assessing possible sex differences in the AUC of each phase using Two-Way ANOVA analysis, no significant differences were noted in either the acute phase (*F*_3,48_ = 2.314, *P* = 0.090) (Supplementary Figure S1C) or in the inflammatory phase (*F*_3,48_ = 0.370, *P* = 0.775) (Supplementary Figure S1D). While AM1241 produced significant acute and inflammatory phase antinociception at both 3 mg/kg and 10 mg/kg, the 10 mg/kg dose produced significantly greater antinociception in the inflammatory phase in male mice (*P* = 0.050) and thus all subsequent experiments involving AM1241 were conducted at that dose.

### ACEA antinociception was independent of 5-HT_1A_ activation

Treatment of C57BL/6J female mice with vehicle, 5-HT_1A_ inhibitor WAY-100635, ACEA 0.5 mg/kg, or ACEA 0.5 mg/kg preceded by WAY-100635 resulted in significant differences between the treatments (*F*_3,20_ = 15.02, *P* < 0.001) that persisted in a time-group dependent manner (*F*_33,220_ = 3.021, *P* = 0.001) (Figure 3A). Multiple comparison analysis revealed that ACEA 0.5 mg/kg significantly reduced pain at 25 min post-formalin injection, relative to both vehicle (*P* = 0.001) and WAY-100635 (*P* = 0.01) treated mice (Figure 3A). Mice that received ACEA preceded by WAY-100635 demonstrated similar antinociceptive effects, with significantly reduced pain at 25 min post-formalin injection relative to vehicle (*P* = 0.03) and WAY-100635 (*P* = 0.050) treated mice (Figure 3A). AUC analysis in the acute phase of pain revealed no significant differences between the treatments (*F*_3,20_ = 1.499, *P* = 0.25) (Figure 3B). Conversely, AUC analysis in the inflammatory phase revealed significant differences between the treatments (*F*_3,20_ = 16.32, *P* < 0.001), with *post-hoc* analysis revealing significantly reduced inflammatory pain following injection with ACEA (relative to vehicle, *P* < 0.001; relative to WAY-100635, *P* = 0.002) or injection with ACEA preceded by WAY-100635 (relative to vehicle, *P* < 0.001; relative to WAY-100635, *P* < 0.001) (Figure 3C).

**Figure 3:**
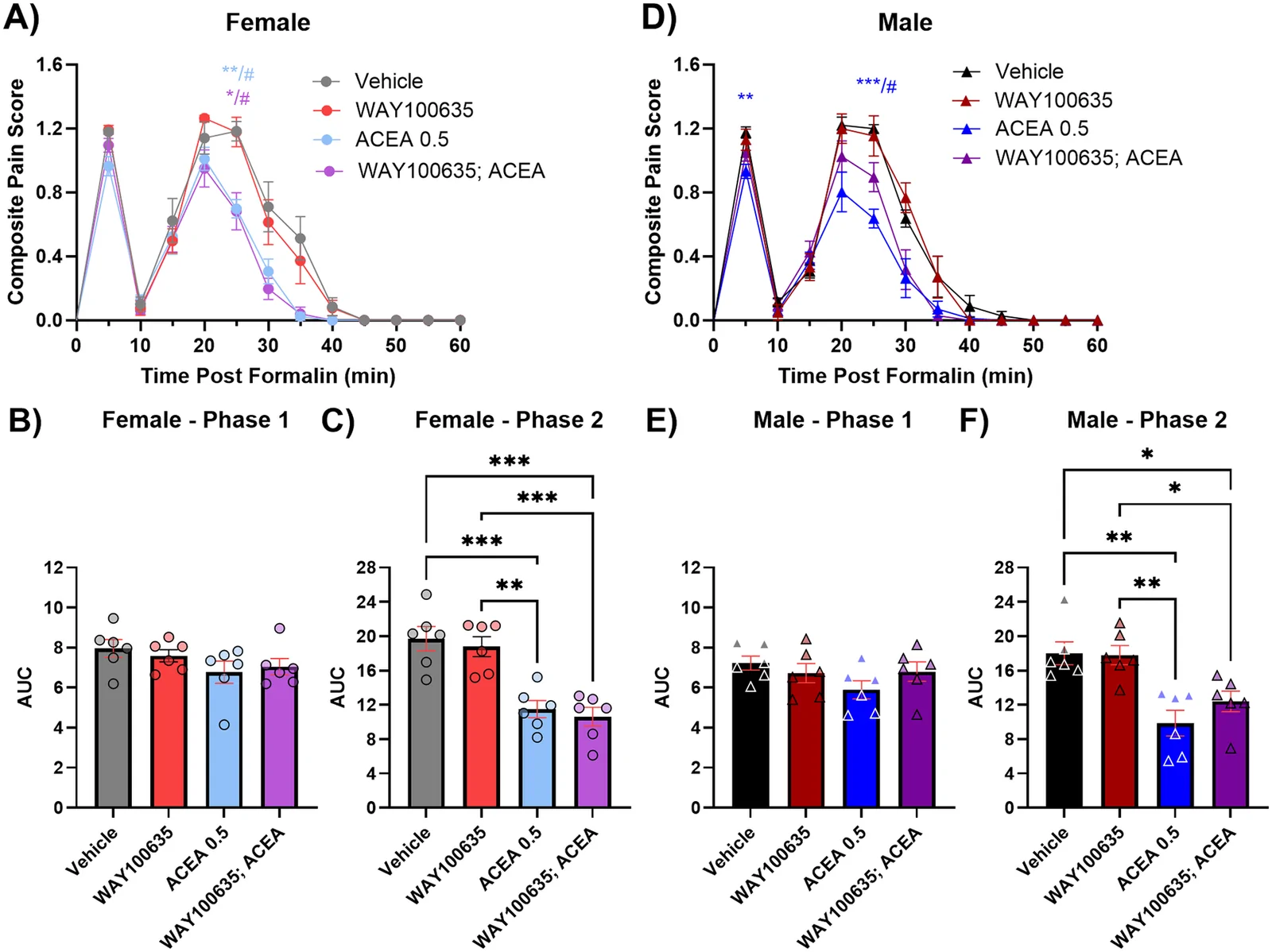
ACEA antinociception was independent of 5-HT_1A_ activation. ACEA (0.5 mg/kg) antinociception was unaffected by prior treatment with WAY-10063 (5-HT_1A_ antagonist) in both female **(A)** and male **(D)** C57BL/6J mice. No significant acute phase antinociception was noted in female **(B)** or male **(E)** mice. In the inflammatory phase, ACEA resulted in significant antinociception, regardless of pretreatment with WAY-100635, in both female **(C)** and male **(F)** mice. Data is expressed as mean ± SEM (*n* = 6 per group). Repeated measures one-way ANOVA with Greenhouse–Geisser correction used to calculate significance for **(A)** and **(D)** One-way ANOVA with Bonferroni *post-hoc* used to calculate significance for **(B)**, **(C)**, **(E)**, and **(F)**. Relative to vehicle: *P < 0.05, **P < 0.01, ***P < 0.001; relative to WAY-100635: ^#^*P* < 0.05, ^##^*P* < 0.01, ^###^*P* < 0.001.

Treatment of C57BL/6J male mice with vehicle, WAY-100635, ACEA 0.5 mg/kg, or ACEA 0.5 mg/kg preceded by WAY-100635 resulted in significant differences between the treatments (*F*_3,20_ = 9.846, *P* < 0.001) that persisted in a time-group dependent manner (*F*_33,220_ = 3.402, *P* < 0.001) (Figure 3D). Subsequent multiple comparison analysis revealed that ACEA 0.5 mg/kg significantly reduced pain at 5 min (relative to vehicle, *P* = 0.010) and 25 min (relative to vehicle, *P* < 0.001; relative to WAY-100635, *P* = 0.04) (Figure 3D). In the acute phase, AUC analysis revealed no significant differences between the treatments (*F*_3,20_ = 1.551, *P* = 0.23) (Figure 3E). AUC analysis in the inflammatory phase revealed significant differences between the treatments (*F*_3,20_ = 9.719, *P* < 0.001), with *post-hoc* analysis revealing that significant antinociceptive effects following injection of ACEA (relative to vehicle, *P* = 0.002; relative to WAY-100635, *P* = 0.002) or ACEA preceded by WAY-100635 (relative to vehicle, *P* = 0.04; relative to WAY-100635, *P* = 0.050) (Figure 3F).

Potential sex differences in the antinociceptive efficacy of ACEA 0.5 mg/kg and the effects of WAY-100635, alone or preceding ACEA, were investigated via Two-Way Repeated Measures ANOVA analysis, with no significant differences noted between the treatments (*F*_33,440_ = 0.744, *P* = 0.732). When analyzing the AUC, no significant sex differences were noted in the acute phase (*F*_3,48_ = 0.224, *P* = 0.879) (Supplementary Figure S1E) or in the inflammatory phase (*F*_3,48_ = 0.888, *P* = 0.456) (Supplementary Figure S1F).

### AM1241 acute phase antinociception occurred via a 5-HT1A-dependent mechanism

Treatment of C57BL/6J female mice with vehicle, 5-HT_1A_ inhibitor WAY-100635, or AM1241 preceded by WAY-100635 resulted in significant differences between the treatments (*F*_3,20_ = 28.94, *P* < 0.001) that persisted in a time-group dependent manner (*F*_33,220_ = 7.702, *P* < 0.001) (Figure 4A). Multiple comparison analysis revealed that AM1241 provided significant antinociception at 5 min relative to both vehicle (*P* < 0.001) and WAY-100635 (*P* = 0.009); this antinociception was prevented by pretreatment with WAY-100635 (AM1241 alone vs. WAY-100635 and AM1241, *P* < 0.001) (Figure 4A). During the inflammatory phase, antinociception was observed in mice treated with either AM1241 alone (relative to vehicle: at 20 min, *P* = 0.01; at 25 min, *P* < 0.001) (relative to WAY-100635: at 20 min, *P* = 0.04; at 25 min, *P* < 0.001; at 30 min, *P* = 0.009; at 35 min, *P* = 0.04) and in mice treated with both AM1241 and WAY-100635 (relative to vehicle: at 20 min, *P* = 0.03; at 25 min, *P* = 0.010) (relative to WAY-100635: at 30 min, *P* = 0.03) (Figure 4A). AUC analysis in the acute phase of pain revealed significant differences between the treatments (*F*_3,20_ = 12.66, *P* < 0.001), with multiple comparison analysis revealing that AM1241 treatment resulted in significantly decreased nociception relative to vehicle (*P* < 0.001), WAY-100635 (*P* = 0.003), and relative to mice that received AM1241 after pretreatment with WAY-100635 (*P* < 0.001) (Figure 4B). These findings represent the reversal of AM1241-induced acute phase antinociception by 5-HT_1A_ inhibition. Similarly, AUC analysis in the inflammatory phase revealed significant differences between the treatments (*F*_3,20_ = 28.47, *P* < 0.001); however, multiple comparison analysis of this phase revealed significant antinociception, relative to vehicle, for both groups that received AM1241 alone (relative to vehicle: *P* < 0.001; relative to WAY-100635: *P* < 0.001) and those that received both AM1241 and pretreatment with WAY-100635 (relative to vehicle: *P* < 0.001; relative to WAY-100635: *P* < 0.001) (Figure 4C).

**Figure 4:**
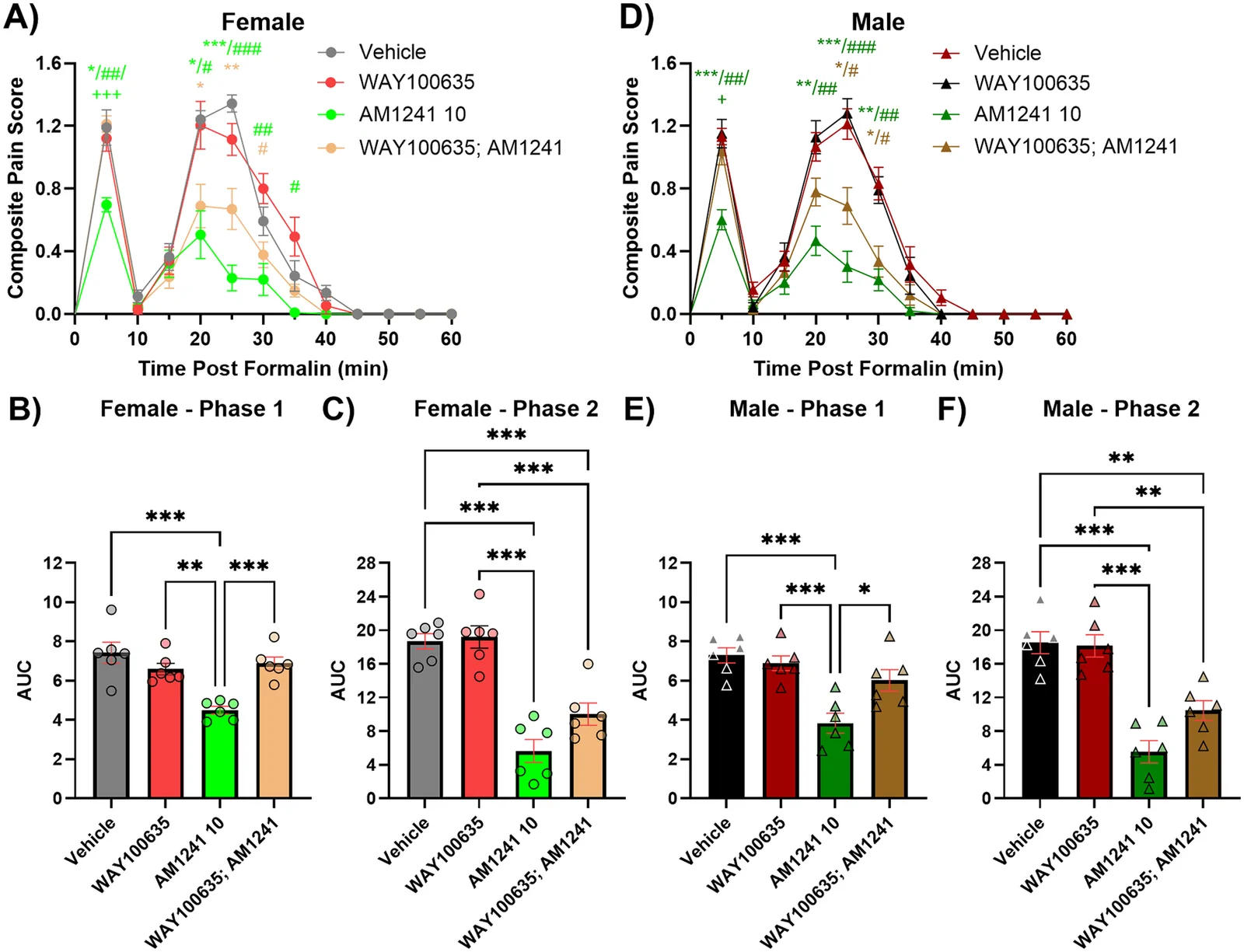
AM1241 acute phase antinociception was dependent on 5-HT_1A_ activation. Treatment of C57BL/6J mice with AM1241 (10 mg/kg) resulted in significant antinociception that was partially reversed by pretreatment with WAY-100635 in both female **(A)** and male **(D)** mice. In the acute phase, the antinociception provided by AM1241 was fully prevented by pretreatment with WAY-100635 in both female **(B)** and male **(E)** mice. In the inflammatory phase, the antinociception provided by AM1241 was unaffected by pretreatment with WAY-100635 in female **(C)** and male **(F)** mice. Data is expressed as mean ± SEM (*n* = 6 per group). Repeated measures one-way ANOVA with Greenhouse–Geisser correction used to calculate significance for **(A)** and **(D)**. One-way ANOVA with Bonferroni *post-hoc* used to calculate significance for **(B)**, **(C)**, **(E)**, and **(F)**. Relative to vehicle: *P < 0.05, **P < 0.01, ***P < 0.001; relative to WAY-100635: ^#^*P* < 0.05, ^##^*P* < 0.01, ^###^*P* < 0.001; relative to WAY-100635 followed by AM1241: ^+^*P* < 0.05, ^++^*P* < 0.01, ^+++^*P* < 0.001.

In male mice treated under similar conditions, significant differences were noted between the treatments (*F*_3,20_ = 24.71, *P* < 0.001) and this effect persisted in a time-group dependent manner (*F*_33,220_ = 7.267, *P* < 0.001) (Figure 4D). Multiple comparison analysis revealed that AM1241 provided significant antinociception at 5 min relative to both vehicle (*P* < 0.001) and WAY-100635 (*P* = 0.004); this antinociception was prevented by pretreatment with WAY-100635 (AM1241 alone vs. WAY-100635 and AM1241, *P* = 0.01) (Figure 4D). During the inflammatory phase, antinociception was observed in mice treated with either AM1241 alone (relative to vehicle: at 20 min, *P* = 0.004; at 25 min, *P* < 0.001; at 30 min, *P* = 0.004) (relative to WAY-100635: at 20 min, *P* = 0.004; at 25 min, *P* < 0.001; at 30 min, *P* = 0.002) and in mice treated with both AM1241 and WAY-100635 (relative to vehicle: at 25 min, *P* = 0.03; at 30 min, *P* = 0.03) (relative to WAY-100635: at 25 min, *P* = 0.01; at 30 min, *P* = 0.03) (Figure 4D). AUC analysis of the acute phase revealed significant differences between the treatments (*F*_3,20_ = 11.31, *P* < 0.001) that subsequent multiple comparison analysis revealing that AM1241 treatment resulted in significantly decreased nociception relative to vehicle (*P* < 0.001), WAY-100635 (*P* < 0.001), and relative to mice that received AM1241 after pretreatment with WAY-100635 (*P* = 0.02) (Figure 4E). These findings represent the reversal of AM1241-induced acute phase antinociception by 5-HT_1A_ inhibition. Inflammatory phase AUC analysis revealed significant differences between the treatments (*F*_3,20_ = 23.84, *P* < 0.001) with multiple comparison analysis revealing significantly reduced pain in mice treated with either AM1241 alone (relative to vehicle, *P* < 0.001; relative to WAY-100635, *P* < 0.001) or with AM1241 preceded by WAY-100635 (relative to vehicle, *P* = 0.002; relative to WAY-100635, *P* = 0.003) (Figure 4F).

Potential sex differences in the antinociceptive efficacy of AM1241 10 mg/kg, with or without pretreatment by 5-HT_1A_ antagonist WAY-100635, were found to be non-significant (*F*_33,440_ = 0.850, *P* = 0.617). When assessing possible sex differences in the AUC of each phase, no significant differences were noted in either the acute phase (*F*_3,48_ = 0.823, *P* = 0.489) (Supplementary Figure S1G) or in the inflammatory phase (*F*_3,48_ = 0.115, *P* = 0.951) (Supplementary Figure S1H).

### ACEA (0.5 mg/kg) and AM1241 (10 mg/kg) did not significantly alter open field locomotion or center duration

Treatment of C57BL/6J female mice with vehicle, 5-HT_1A_ inhibitor WAY-100635, ACEA 0.5 mg/kg, ACEA 0.5 mg/kg preceded by WAY-100635, AM1241 10 mg/kg, or AM1241 preceded by WAY-100635, produced no significant locomotion differences between the treatments (*F*_5,30_ = 0.9972, *P* = 0.44) and so significant time × group dependent differences (*F*_20,120_ = 1.700, *P* = 0.10) (Figure 5A). No significant differences were noted in baseline open field locomotion (*F*_5,30_ = 0.7258, *P* = 0.61) and controlling for baseline open field locomotion yielded no significant differences in the assessed interaction (*F*_15,90_ = 0.9937, *P* = 0.46) (Supplementary Figure S2A). Assessment of these same mice for the center duration during the open field test revealed no significant differences between the treatments (*F*_5,30_ = 2.266, *P* = 0.07) and no significant time × group dependent differences (*F*_20,120_ = 1.969, *P* = 0.06) (Figure 5C). No significant differences were noted in baseline open field center duration (*F*_5,30_ = 2.449, *P* = 0.06) and controlling for baseline open field center duration yielded no significant differences in the assessed interaction (*F*_15,90_ = 1.857, *P* = 0.10) (Supplementary Figure S2C).

**Figure 5:**
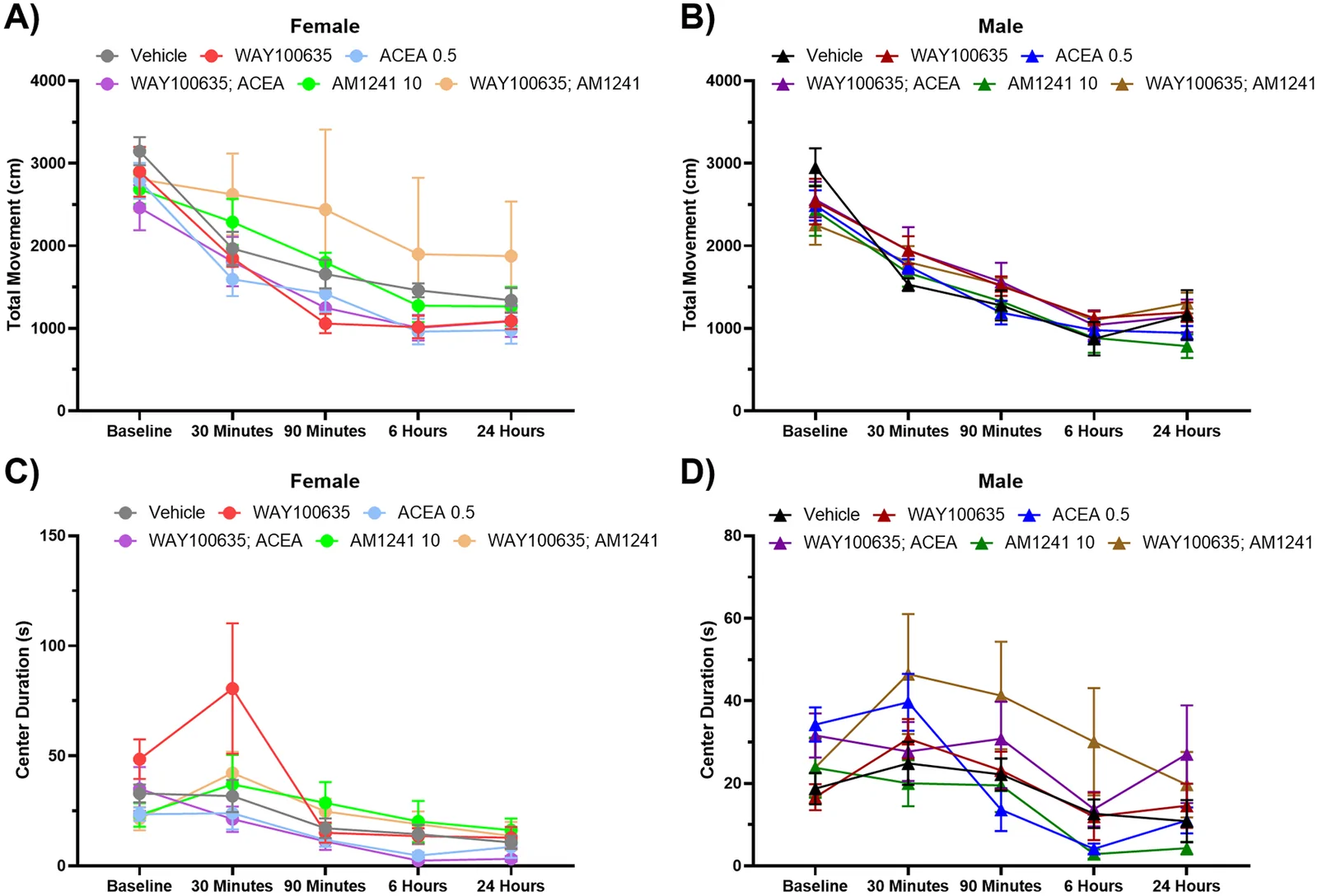
ACEA and AM1241 did not significantly alter open field performance. Open field locomotion and center duration were assessed in female and male C57BL/6J mice. Administration of ACEA, AM1241, WAY-100635, and their combinations resulted in no significant differences in locomotion in either female **(A)** or male **(B)** mice. Similarly, no significant differences in center duration were noted in either female **(C)** or male **(D)** mice. Data is expressed as mean ± SEM (*n* = 6 per group). Repeated measures one-way ANOVA with Greenhouse–Geisser correction used to calculate significance for **(A)**, **(B)**, **(C)**, and **(D)**.

Male C57BL/6J mice treated under the same conditions produced no significant locomotion differences between the treatments (*F*_5,30_ = 0.4262, *P* = 0.83) though significant time × group dependent differences were noted (*F*_20,120_ = 1.807, *P* = 0.04) (Figure 5B). No significant differences were noted in baseline open field locomotion (*F*_5,30_ = 0.8813, *P* = 0.51) and controlling for baseline open field locomotion yielded no significant differences in the assessed interaction (*F*_15,90_ = 0.8294, *P* = 0.62) (Supplementary Figure S2B). Subsequent multiple comparison analysis revealed no significant comparisons between any two treatments. Assessment of these same mice for differences in open field center duration similarly revealed no significant differences between the treatments (*F*_5,30_ = 1.488, *P* = 0.22), significant time × groups dependent effects (*F*_20,120_ = 1.949, *P* = 0.02), and no significant comparisons upon subsequent multiple comparison analysis (Figure 5D). No significant differences were noted in baseline open field center duration (*F*_5,30_ = 1.685, *P* = 0.17) and controlling for baseline open field center duration yielded no significant differences in the assessed interaction (*F*_15,90_ = 1.311, *P* = 0.23) (Supplementary Figure S2D).

### ACEA (0.5 mg/kg) and AM1241 (10 mg/kg) did not significantly alter rotarod locomotion/motor coordination

Treatment of female mice with vehicle, 5-HT1A inhibitor WAY-100635, ACEA 0.5 mg/kg, ACEA 0.5 mg/kg preceded by WAY-100635, AM1241 10 mg/kg, or AM1241 preceded by WAY-100635, failed to produce significant differences between the treatments (*F*_5,29_ = 2.069, *P* = 0.10) and failed to produce significant time × group dependent differences (*F*_20,116_ = 1.463, *P* = 0.15) (Figure 6A). No significant differences were noted in baseline rotarod performance (*F*_5,29_ = 2.070, *P* = 0.10) and controlling for baseline rotarod performance yielded no significant differences in the assessed interaction (*F*_15,87_ = 1.800, *P* = 0.08) (Supplementary Figure S3A). When assessing male mice treated with the same compounds, significant differences were noted between the treatments (*F*_5,29_ = 12.71, *P* < 0.001) that failed to persist in a time-group dependent manner (*F*_20,116_ = 1.252, *P* = 0.26) (Figure 6B). Significant differences were noted in baseline rotarod performance (*F*_5,29_ = 10.96, *P* < 0.001) and controlling for baseline rotarod performance yielded significant differences in the assessed interaction (*F*_15,87_ = 2.261, *P* = 0.01) (Supplementary Figure S3B). However, subsequent multiple comparison analysis revealed no significant comparisons between the treatments (Supplementary Figure S3B).

**Figure 6:**
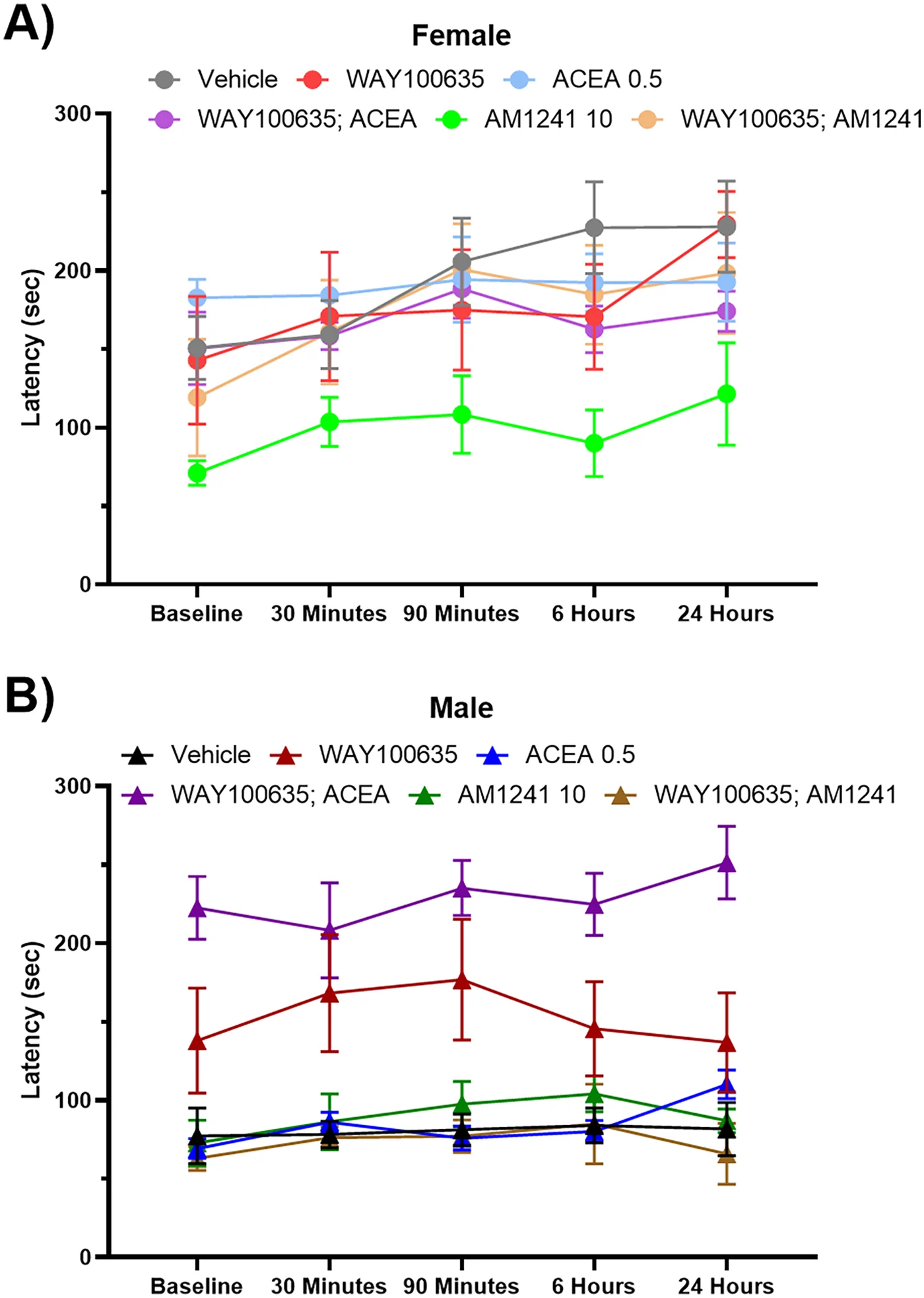
ACEA did not significantly alter rotarod performance. Treatment of female and male C57BL/6J mice with ACEA, AM1241, WAY-100635, and their combinations resulted in no significant differences in rotarod locomotion/motor coordination in female **(A)** or male **(B)** mice. Data is expressed as mean ± SEM (*n* = 6 per group). Repeated measures one-way ANOVA with Greenhouse–Geisser correction used to calculate significance for **(A)** and **(B)**.

### ACEA (0.5 mg/kg) and AM1241 (10 mg/kg) did not significantly alter rectal temperature

Treatment of C57BL/6J female mice with vehicle, 5-HT1A inhibitor WAY-100635, ACEA 0.5 mg/kg, ACEA 0.5 mg/kg preceded by WAY-100635, AM1241 10 mg/kg, or AM1241 preceded by WAY-100635, resulted in significant differences in rectal temperature between the treatments (*F*_5,30_ = 3.372, *P* = 0.02) that failed to persist in a time-group dependent manner (*F*_20,120_ = 0.7243, *P* = 0.77) (Figure 7A). No significant differences were noted in baseline rectal temperature (*F*_5,30_ = 0.8565, *P* = 0.52) and controlling for baseline rectal temperature yielded no significant differences in the assessed interaction (*F*_15,90_ = 0.5684, *P* = 0.87) (Supplementary Figure S4A). Treatment of C57BL/6J male mice with vehicle, 5-HT1A inhibitor WAY-100635, ACEA 0.5 mg/kg, ACEA 0.5 mg/kg preceded by WAY-100635, AM1241 10 mg/kg, or AM1241 preceded by WAY-100635, similarly resulted in significant differences in rectal temperature between the treatments (*F*_5,30_ = 3.578, *P* = 0.01) that failed to persist in a time-group dependent manner (*F*_20,120_ = 1.507, *P* = 0.13) (Figure 7B). Significant differences were noted in baseline rectal temperature (*F*_5,30_ = 3.511, *P* = 0.01); controlling for baseline rectal temperature, however, yielded no significant differences in the assessed interaction (*F*_15,90_ = 1.744, *P* = 0.09) (Supplementary Figure S4B).

**Figure 7:**
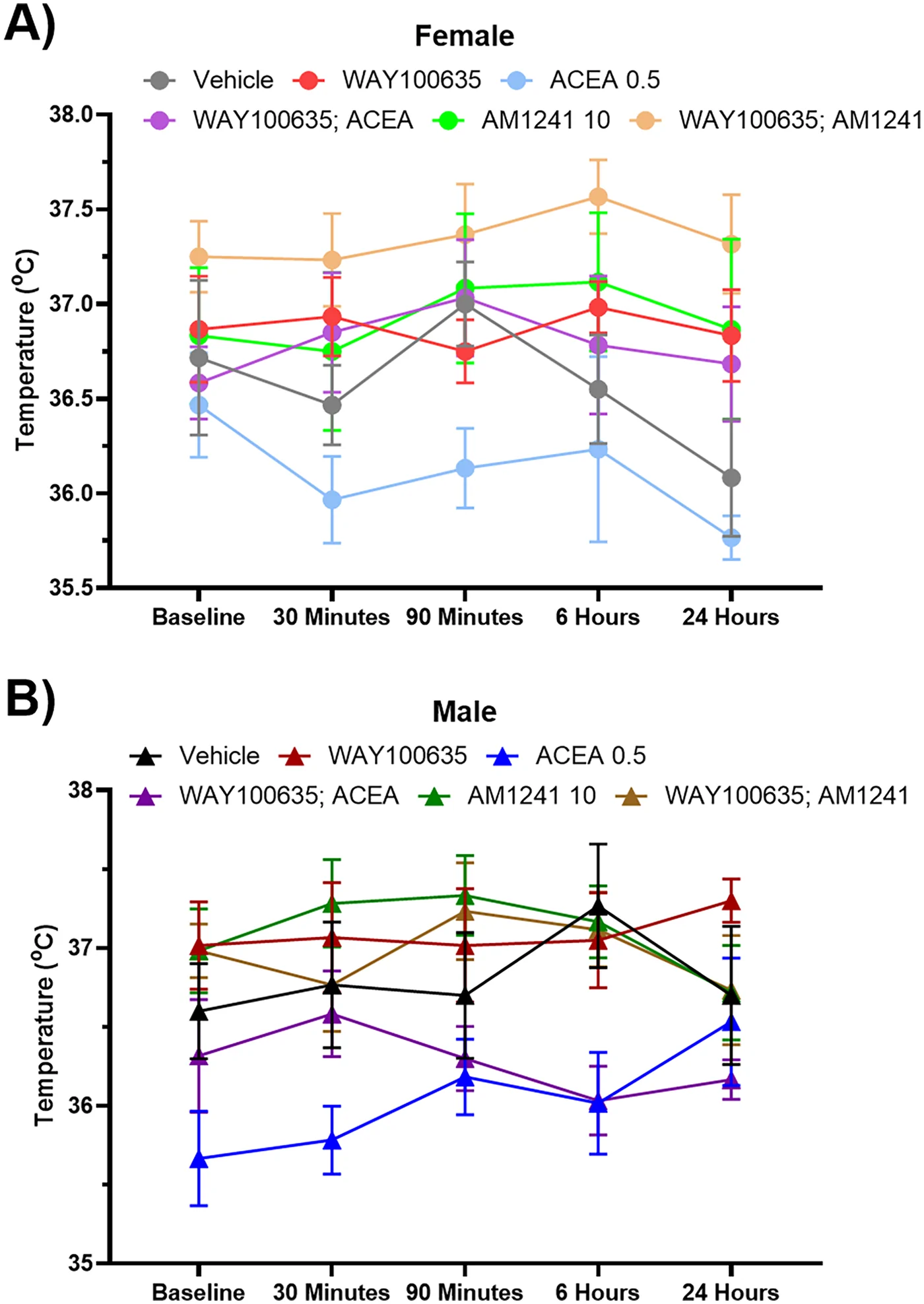
ACEA did not significantly alter rectal temperature. Treatment of female and male C57BL/6J mice with ACEA, AM1241, WAY-100635, and their combinations resulted in no significant differences in rectal temperature in female **(A)** or male **(B)** mice. Data is expressed as mean ± SEM (*n* = 6 per group). Repeated measures one-way ANOVA with Greenhouse–Geisser correction used to calculate significance for **(A)** and **(B)**.

## Discussion

Inflammatory pain is a key characteristic of acute traumatic pain and chronic rheumatic disease, and a significant portion of patients suffer from these pain conditions without adequate pain management (1, 8, 56). Complicating the management of pain is the reality that the majority of afflicted patients are women while the majority of pain research has been male-focused (12–16). The endocannabinoid system represents a key modulator of pain and, as such, cannabinoid targeting compounds represent an important target in pain management (21, 23, 25, 57). Research has repeatedly demonstrated that activation of either the CB_1_ or CB_2_ receptor can provide significant antinociception in preclinical pain models (24, 27–30). Recent research has revealed a functional connection between the 5-HT_1A_ receptor and the endocannabinoid system through the CB_2_ receptor (42, 43). As chronic pain conditions severely decrease a patient's quality of life and their prevalence is rising, the discovery of novel analgesics is of significant clinical interest (9, 58, 59).

In this study, we demonstrated that the CB_1_ selective agonist ACEA produces significant antinociception in the formalin model of inflammatory pain. Notably, there were significant dose effects of ACEA, with the 0.5 mg/kg dose producing the strongest inflammatory phase antinociception in both male and female mice. While ACEA is considered a selective agonist of CB_1_ receptors, which produce antinociceptive effects when activated, several studies have previously suggested that ACEA produces off-target activation of transient receptor potential vanilloid 1 (TRPV1) receptors (60, 61). TRPV1 activation is associated with inflammation and hyperalgesia, and spinal TRPV1 receptors have previously been shown to play a role in the transduction of formalin-induced pain (62, 63). It is possible that the loss of significant antinociception at higher doses of ACEA are due to the pro-nociceptive activation of TRPV1 receptors offsetting any antinociceptive benefit of CB_1_ receptor activation. Further research should investigate the relative activation of CB_1_ vs. TRPV1 receptors at differing doses of ACEA. Additionally, previous studies have noted a similar biphasic antinociceptive and anxiolytic effect of CB_1_ activation, with the biphasic anxiolytic effect having been attributed to the differential activation of CB_1_ receptors on glutaminergic neurons at low doses (anxiolytic) while high doses activated CB_1_ receptors on GABAergic neurons (64–66). Future studies should investigate whether differential activation of CB_1_ receptors on differing neuron populations similarly plays a role in the observed antinociceptive biphasic dose-response curve.

There were also significant sex differences in the effects of ACEA, with male mice having statistically significant acute and inflammatory phase antinociception at a dose of ACEA (0.75 mg/kg) that produced neither of these effects in female mice. Similar to the endocannabinoid ligand anandamide, ACEA is metabolized by fatty acid amide hydrolase (67). Previous studies have demonstrated that estradiol and progesterone regulate both the expression and activity of fatty acid amide hydrolase (68, 69). While cytochrome P450 enzymes are known to contribute to the metabolism of phytocannabinoids, with significant sex differences in their activity, research into their metabolism of synthetic cannabinoid ligands is limited and without account to possible sex-specific differences in metabolism (70, 71). Further research investigating the metabolism and possible biotransformation of commonly used synthetic cannabinoids is needed. It has previously been established that there are significant preclinical sex differences in the endocannabinoid system and the antinociceptive efficacy of cannabinoid agonists (23). With regards to ACEA and CB_1_ agonism, it has been demonstrated that there are significant sex differences in both brain region-specific receptor densities and in the affinity of these CB_1_ receptors for cannabinoid-based drugs (72, 73). A recent study attributed sex differences in ACEA-mediated antinociception, in a murine model of neuropathic pain, to sexually dimorphic expression of CB_1_ receptors on CaMKIIα neurons in medial prefrontal cortex (72). Further studies are needed to confirm if humans similarly demonstrate significant sex differences in CB_1_ receptor density, affinity, and efficacy.

The CB_2_-selective agonist AM1241 produced significant dose-dependent antinociception in both the acute and inflammatory phases of the formalin test. These findings are similar to previous research, which has demonstrated a significant antinociceptive benefit of CB_2_ agonism through AM1241 in murine models of acute, inflammatory, thermal, and neuropathic pain (24, 29, 74, 75). As CB_2_ activation resulted in antinociception without notable sex differences, and as it is not associated with the psychoactive side effects of CB_1_ activation, further investigation is needed to evaluate the clinical potential of CB_2_-selective analgesics (47, 76). Additionally, AM1241 has previously been found to produce off-target, indirect activation of *μ*-opioid receptors through stimulation of β-endorphin release from keratinocytes (77, 78). Some studies have noted that the antinociceptive effects of AM1241 may be partially reversed by the opioid antagonist naloxone (77, 79) while others have demonstrated that AM1241 produces antinociception that is unaffected by naloxone (46). It is possible that this off-target indirect activation of opioid signaling contributed to AM1241 producing acute phase antinociception while ACEA only did so at 0.75 mg/kg in male mice. Additionally, CB_2_ agonism has previously been demonstrated to decrease inflammation through inhibition of mast cells and decreased neutrophilic accumulation (80, 81). This anti-inflammatory effect may also contribute to the significant acute phase antinociception observed with AM1241, as mast cell activation has been previously demonstrated to sensitize TRPA1 channels that play a key role in the acute phase of formalin-induced pain (50, 82). Further studies should investigate the role of opioid and inflammatory signaling in AM1241-induced antinociception within the formalin test of inflammatory pain.

The 5-HT_1A_ receptor has emerged as a promising target for potential analgesic and anti-depressive medications (36, 37). Due, in part, to the severe side effects associated with opioid-based medications, current guidelines recommend using a multimodal approach to pain management that combines non-steroid anti-inflammatories, anticonvulsants, and anti-depressants to manage pain (18). Antidepressants, including tricyclic antidepressants and selective norepinephrine reuptake inhibitors, have previously been shown to provide significant antinociceptive benefit in several pain conditions (83, 84). These compounds primarily provide analgesia through enhanced noradrenergic signaling at α_2A_-adrenoceptors and enhanced serotoninergic signaling at 5-HT_1A_ receptors (85–87). The 5-HT_1A_-selective partial agonist buspirone is an anxiolytic compound that has also shown benefit in the management of depression both alone and as an adjunct to treatment with selective serotonin reuptake inhibitors (88–90). Buspirone has been shown in preclinical trials to provide significant antinociceptive benefit when given chronically or at high doses (91–93). While clinical findings have been largely non-significant, the experiments were limited to either a single dose (94, 95) or repeated low doses (96) due to a concern detrimental side effects. In our study, we found that the 5-HT_1A_ antagonist WAY-100635 did not significantly alter nociception relative to vehicle treated mice. This is consistent with previous findings, which identified WAY-100635 as a neutral, or “silent,” antagonist of the 5-HT_1A_ receptor (97, 98). As a “silent” antagonist, WAY-100635 binds to the 5-HT_1A_ receptor without triggering any cellular response while preventing the binding of either agonists or inverse agonists to the receptor (99).

Recent studies have connected the 5-HT_1A_ receptor both to the activity of the phytocannabinoid cannabidiol and to CB_2_-dependent activity (41–43). Our findings lend further support to a functional connection between the CB_2_ and 5-HT_1A_ receptor. We found that blockade of the 5-HT_1A_ receptor eradicated CB_2_-dependent acute phase antinociception, in both male and female mice. No connection between the 5-HT_1A_ receptor and CB_1_ receptor was found in our nociceptive testing. Future studies should explore the use of this selective agonist in combination with a cannabinoid-based compound to enhance its efficacy. Similarly, future studies should explore the benefits of cannabinoid-based compounds in enhancing antidepressant-dependent antinociception and in enhancing the anti-depressive and anxiolytic benefits of buspirone. While our study did not demonstrate any effect of 5-HT_1A_ blockage on CB_2_-mediated antinociception in the inflammatory phase, it is possible that this lack of effect was due to changing circulation concentration of WAY-100635, which is known to have a relatively rapid half-life (100). As WAY-100635 has previously been proven safe at up to 3 mg/kg, further studies should further investigate the functional link between 5-HT_1A_ and CB_2_ receptors in antinociception at higher doses or with a shorter interval between injections (99, 101).

Compounds that activate the CB_1_ receptor in rodents produce the classical cannabinoid tetrad of behavioral effects: hypo-locomotion, hypothermia, catalepsy, and analgesia (102). In our study, the comparatively low dose of ACEA (0.5 mg/kg) allowed for antinociceptive effects to occur without statistically significant changes in locomotion, temperature, or catalepsy. Additionally, neither the CB_2_ selective agonist AM1241 (10 mg/kg) nor the 5-HT_1A_ antagonist WAY-100635 resulted in significant alterations in locomotion, temperature, or catalepsy.

Critically, our findings demonstrated no evidence that the treatments used, injections, or testing schedule produced significant stress or impaired thermoregulation. Stress has previously been shown to produce an analgesic effect that could limit the interpretation of pain behavioral testing results (103). As stress is associated in mice with an anxiety-like phenotype that would increase peripheral region preference in the open field test, it is safe to conclude that the mice tested here were not in significant stress as none of the treatments demonstrated significant changes in their center time duration in the open field (104, 105). Similarly, hypothermia has been shown to produce an analgesic effect in murine models of pain (106, 107). As none of the compounds tested significantly altered rectal temperature, this allows for further confidence in the validity of our antinociceptive findings.

These findings support the growing interest in the use of cannabinoid-based compounds for the management of acute and inflammatory pain, the importance of investigating sex differences, and the presence of a functional connection between the CB_2_ and 5-HT_1A_ receptors (23, 41–43, 108). The significant sex differences in the efficacy of ACEA in the formalin test of inflammatory pain warrant further investigation in murine models of chronic inflammation and into whether these effects directly translate to women using cannabinoid-based compounds. Additionally, as most pain conditions are chronic rather than acute, future research should be conducted to evaluate the long-term development of tolerance to the effects of CB_1_- and CB_2_-based analgesics in preclinical models. These results support the need for further investigation into functionally and clinically relevant connections between the endocannabinoid system, specifically the CB_2_ receptor, and the 5-HT_1A_ receptor. Further studies are needed to determine the extent of this connection, including whether it plays a role in the non-antinociceptive effects of CB_2_ activation and to confirm its presence in humans. While further research is needed, these potential combinations represent a promising future area of exploration in the field of chronic pain management.

## Data Availability

The original contributions presented in the study are included in the article/Supplementary Material, further inquiries can be directed to the corresponding author.
