# Supplementary material for: Assessing CB1 and CB2 receptor agonist antinociception while exploring sex differences and 5-HT1A-dependent mechanisms
Source: Front Pain Res (Lausanne). 2026 Oct 26;7:2005000. doi: 10.3389/fpain.2026.2005000 (PMC13745691; doi:10.3389/fpain.2026.2005000)
Supplement: Supplementary file 1 [file Datasheet1.docx]

Supplementary Material

**Supplementary Figure 1.** Assessment of antinociceptive sex differences. C57BL/6J mice demonstrated significant sex differences in their dose response to ACEA, with male mice demonstrating increased antinociceptive benefit for 0.75 mg/kg dose in both the **Acute Phase (A)** and **Inflammatory Phase (B)** of the formalin test. No significant sex differences were noted in the dose response to AM1241 in either the **Acute Phase (C)** or **Inflammatory Phase (D)**. No significant sex differences were noted in the effects of WAY-100635 pretreatment on the antinociceptive effects of ACEA**,** in either the **Acute Phase (E)** or **Inflammatory Phase (F)**. No significant sex differences were noted in the effects of WAY-100635 on AM1241 mediated antinociception in either the **Acute Phase (G)** or **Inflammatory Phase (H)**. Data is expressed as mean ± SEM (n=6 per group). Significance calculated using two-way ANOVA for **(A)**, **(B)**, **(C)**, **(D)**, **(E)**, **(F)**, **(G)**, and **(H)**. Relative to treatment-matched group (e.g. female vehicle vs male vehicle): @p<0.05, @@p<0.01, @@@p<0.001.

**Supplementary Figure 2.** ACEA and AM1241 did not significantly alter open field performance after accounting for baseline differences. Open field locomotion and center duration were assessed in female and male C57BL/6J mice. Administration of ACEA, AM1241, WAY-100635, and their combinations resulted in no significant differences in locomotion in either **female (A)** or **male (B)** mice. Similarly, no significant differences in center duration were noted in either **female (C)** or **male (D)** mice. Data is expressed as mean ± SEM (n=6 per group). Repeated measures one-way ANOVA with Greenhouse-Geisser correction used to calculate significance for **(A), (B), (C),** and **(D)**.

**Supplementary Figure 3.** ACEA did not significantly alter rotarod performance after accounting for baseline differences. Treatment of female and male C57BL/6J mice with ACEA, AM1241, WAY-100635, and their combinations resulted in no significant differences in rotarod locomotion/motor coordination in **female (A)** mice. In **male (B)** mice treated under the same conditions, there was a statistically significant main effect but no statistically significant multiple comparisons were noted on multiple comparison analysis. Data is expressed as mean ± SEM (n=6 per group). Repeated measures one-way ANOVA with Greenhouse-Geisser correction used to calculate significance for **(A)** and **(B).**

**Supplementary Figure 4.** ACEA did not significantly alter rectal temperature after accounting for baseline differences. Treatment of female and male C57BL/6J mice with ACEA, AM1241, WAY-100635, and their combinations resulted in no significant differences in rectal temperature in **female (A)** or **male (B)** mice. Data is expressed as mean ± SEM (n=6 per group). Repeated measures one-way ANOVA with Greenhouse-Geisser correction used to calculate significance for **(A)** and **(B).**
